# Prevalence of chronic HBV infection in pregnant woman attending antenatal care in a tertiary hospital in Mwanza, Tanzania: a cross-sectional study

**DOI:** 10.1186/s12879-020-05096-2

**Published:** 2020-06-05

**Authors:** Karin Geffert, Tongai G. Maponga, Shimba Henerico, Wolfgang Preiser, Stella Mongella, August Stich, Samuel Kalluvya, Andreas Mueller, Christa Kasang

**Affiliations:** 1 Medical Mission Institute, Wuerzburg, Germany; 2 Division of Medical Virology, University of Stellenbosch, Faculty of Medicine and Health Sciences, Francie van Zijl Avenue, Tygerberg Cape Town, South Africa; 3 Bugando Medical Centre, Mwanza, Tanzania; 4 Catholic University of Health and Allied Health Sciences, Mwanza, Tanzania

**Keywords:** Hepatitis B, Pregnancy, Tanzania, Vertical transmission

## Abstract

**Background:**

Tanzania has a high prevalence (7.17%) of chronic hepatitis B infection. Mother to Child transmission is very common, resulting in high rate of chronic infections. Currently, there is no screening program for HBV in pregnant women. This study investigated the prevalence and risk factors for chronic HBV infection in pregnant women in a tertiary hospital in Mwanza, Tanzania.

**Methods:**

Seven hundred and forty-three women attending antenatal care and/or delivering at the Bugando Medical Centre were enrolled. All answered a questionnaire on sociodemographic and other risk factors and were tested for HBsAg using a rapid test. In HBsAg positive mothers, maternal blood and umbilical cord blood samples collected after delivery were analyzed for serological (HBsAg, HBeAg and anti-HBe) and virologic (HBV-DNA viral load and genotype) markers. All their babies were vaccinated within 24 h of delivery. The children were followed up at 3 years of age. Data was analyzed using the Mann-Whitney U-test, independent sample T-test and logistic regression.

**Results:**

Of the 743 participants, 22 (3%) were positive for HBsAg, and 2 (9%) had detectable HBe-antigen. Low condom use was the only statistically significant risk factor for chronic HBV infection (OR = 3.514, 95%CI = 1.4–8.0). Of 14 maternal blood samples genotyped, 10 (71%) were genotype A and 4 (29%) were genotype D. HBV-DNA was detected in 21/22 samples, with a median of 241 IU/ml (range: 27.4–25.9 × 10^7^ IU/ml). Five (33%) of 15 available cord blood samples were positive for HBsAg and 10 (67%) were negative. At follow-up, one child showed chronic HBV infection characteristics, one had anti-HBs level of 7 mIU/ml and 5/7(71%) had protective anti-HBs levels (> 10 mIU/ml).

**Conclusion:**

This cohort of pregnant women showed a lower-intermediate prevalence of HBV of 3%. In the 3 years follow-up only 1 out of 7 children showed evidence of chronic HBV infection. The child’s mother with high viral load (25.9 × 10^7^ IU/ml), was positive for HBeAg with a high degree of sequence similarity suggesting vertical transmission. These results highlight a need for improved diagnosis and treatment of HBV infection in pregnant women in Tanzania, in order to prevent vertical transmission.

## Background

Around 257 million people worldwide are thought to carry chronic hepatitis B virus (HBV) infection [1]. Although HBV infection is preventable by vaccination, the burden of chronic hepatitis B remains high. The “Global Burden of Disease Study” found an overall increasing trend in disability adjusted life years (DALYS) due to the long-term sequelae of chronic hepatitis B (CHB), which is in contrast to the general trend of decreasing burden from other infectious diseases [2]. Projections indicate that CHB may lead to additional 20 million deaths between 2015 and 2030 [3].

The highest prevalence of HBV infection is found in the Western Pacific Region (6.2%), followed by the African Region (6.1%) [1]. The prevalence of hepatitis B in Tanzania varies from 3.8 to 8.0% according to different studies and cohorts. A systematic review by Schweitzer et al. estimates that the prevalence in Tanzania is higher intermediate with overall 7.2% [4].

Recent studies on hepatitis B in pregnant women in Tanzania showed HBV prevalence ranging from 3.8% in a study in a district hospital in Mwanza [5], 3.9% in a tertiary hospital in Dar es Salaam [6], 4.2% in a primary health center in Moshi [7] to 8.03% in a municipal health facility in Dar es Salaam [8].

In countries with high endemicity of CHB (≥8%) the predominant routes of transmission are perinatal (> 20%) and early childhood infection (> 60%). By contrast, in countries with low HBV endemicity (< 2%) adolescent and adult infections are very common (70–90%), indicating a role for sexual transmission [9]. The risk of developing chronic infection decreases with age: children infected in their first year have a high risk (80–90%), which decreases to 30–50% in those before the age of 6 and to less than 5% in healthy adults [1].

Immunization is the cornerstone of effective prevention for HBV transmission [1]. Vaccination with a 95% efficacy has been available since 1982. In 2002, Tanzania implemented HBV vaccination for children in the 4th, 8th and 12th week after delivery as part of the extended program on immunization (EPI) [10]. Data published by the WHO indicate a 97% coverage of three doses of hepatitis B vaccination in 2017 in Tanzania [11]. However, low rates of HBs antibodies have been observed in children [12, 13]. A hepatitis B vaccine birth dose has not been implemented yet [14].

In resource-constrained settings recommended procedures and diagnostics for the prevention of the mother to child transmission (MTCT) of HBV are only partially available for women due to the costs and lack of logistics: the current situation in sub-Saharan Africa (SSA) mirrors these deficiencies [15]. The application of hepatitis B immunoglobulin is not feasible in a setting where general HBV testing of pregnant women is not in place and also because of the high costs and the limited availability at primary health care centers [16].

Instead, a cost-effective alternative is the identification of women at risk by rapid tests, and the administration of the first dose of the HBV vaccine to the newborn within 24 h of birth [15, 17, 18]. Rapid tests to detect HBsAg in pregnant women have been proven reasonable in detecting HBV infection in low-resource settings in terms of accuracy, reliability, acceptance and performance [19]. Antiviral therapy with tenofovir in the later state of pregnancy further reduces the risk of transmission and is recommended in regions were HBV viral load testing is feasible [20]. Despite growing evidence on the effectiveness of this intervention, the current WHO guidelines do not recommend the use of antiviral therapy to prevent MTCT. This is because intrauterine transmission occurs comparably rarely and studies were unable to show the cost-effectiveness [21].

This study aimed to understand the prevalence of HBV infection in pregnant women in Mwanza, Tanzania, the serological and virologic nature of the infection, as well as identify possible risk factors.

## Methods

### Study design and setting

The cross-sectional study was conducted between October 2014 and March 2015 in the Department of Obstetrics and Gynaecology at Bugando Medical Medical Centre (BMC) in Mwanza, Tanzania at the southern shore of Lake Victoria. The follow-up of the children of HBsAg positive women was conducted in February 2018. The BMC is the second-largest hospital in Tanzania with 1000 beds taking care of 13 million people as the referral hospital of the Lake Zone in Tanzania. It is also a university teaching hospital with an affiliated medical college.

### Study population

All women who were about to give birth at the Bugando Medical Centre were asked to participate in the study. Additionally, women attending the antenatal care clinic were asked to participate. Participation was voluntary and without payment. Inclusion criteria were pregnancy and 18 years of age or older. Exclusion criteria were mental disorders.

### Benefit for the study participant

Directly after birth, infant vaccination is one of the most effective methods to reduce HBV transmission from the infected mother to her child. Pregnant women giving birth at the BMC got the opportunity to be tested for free and, if positive, the HBV vaccine birth dose and follow-up screening for the child were offered free of charge.

At the time of the study, antiviral treatment for HBV-infection using tenofovir was not yet licensed for the treatment of HBV in Tanzania. Trained personnel were available to inform the women about the disease and opportunities of secondary prevention. An information sheet in Kiswahili including relevant facts about the disease and contact address for further follow-up was handed over.

### Assessment of socio-demographic data

A midwife collected information with a standardised questionnaire about self-reported HIV status, age, residence, marital status, level of education, occupation, parity and gravity, history of life-time sexual partners, age at first sexual contact, history of risk factors (blood transfusion, surgical operation, drug use, intravenous drug injection), HBV vaccination, history of sexually-transmitted infections (STIs), condom use, female genital mutilation, sharing of toothbrush or razors and history of jaundice in their family or themselves. The patient data were collected at the time of the enrolment in a case report form (CRF). A copy remained at the investigator’s files. CRF and all original data were readily available for review during scheduled monitoring visits.

### HBV analysis

Testing of pregnant women was performed using a point-of-care HBsAg test (SureScreen, Derby, United Kingdom). According to the manufacturer, sensitivity, specificity and accuracy of the test are > 99% with a cut-off value of 1 ng/mL HBsAg. Midwives were instructed in the use of the HBsAg test and their performance was monitored for 1 week by supervision and accompaniment during the testing and interview. Umbilical cord blood sampling of the HBsAg positive mothers was conducted by the midwives directly after birth. To avoid contamination the first syringe was discarded and trans-placental puncture was not recommended [22].

Plasma samples were frozen at − 20 °C for 6 months before shipping to the Division of Medical Virology at Stellenbosch University in Cape Town, South Africa for further serologic and molecular testing. HBsAg status was confirmed using the Murex HBsAg Version 3 kit (Murex Biotech, Kent, England). HBeAg and anti-HBe were assessed using DiaSorin ETI-EBK PLUS and ETI-AB-EBK PLUS (DiaSorin, Salugia, Italy) respectively.

HBV DNA was extracted from plasma using the QIAamp MinElute Virus Spin kit (QIAGEN, Hilden, Germany). Quantitative polymerase chain reaction (PCR) was performed on the Rotor-Gene 6000 according to a previously described protocol with a lower limit of quantification of 20 IU/ml [23]. Briefly, quantification of HBV DNA was performed on the Bio-Rad CFX96 real-time PCR detection system (Bio-Rad Laboratories, Hercules, CA) using the WHO HBV DNA quantification standard (National Institute of Biological Standards and Controls, Herts, UK) with a viral load of 1 × 10^6^ IU/ml. Samples with detectable HBV DNA were genotyped and sequenced across the HBsAg gene and the overlapping polymerase as previously described on the ABI-Prism-3130xl genetic analyzer (Applied Biosystems, Foster, CA) using the remaining extract from the quantitative HBV DNA detection assay [24]. A nested PCR was performed using the primers listed in Supplementary Table 1. Presence of product was visualized using gel electrophoresis. The PCR products were cleaned up to remove and underwent a sequencing reaction using the BigDye Terminator v3.1 Cycle Sequencing Kit (Applied Biosystems, California, USA). Sequencing reactions were then cleaned up using BigDye XTerminator Purification Kit (Applied Biosystems, California) in order to remove unincorporated terminators and before being loaded on the genetic analyzer. Base calling was performed using Sequencher and contiguous sequences exported. The genotypes of the study participants were derived by submitting the contiguous sequences onto online Geno2pheno HBV subtyping database (https://hbv.geno2pheno.org/index.php) of the Max Plancks Institute of Informatics (Saarbrücken, Germany).

Furthermore, viral sequence alignment was done using ClustalW and phylogeny was inferred by using the Neighbor Joining method based on the Tamura-Nei model. Using MEGA6 software [25]. Reference sequences deposited on GenBank were downloaded and used in the alignment and phylogenetic analysis. Sequences derived from this study have been submitted onto GenBank (MN558930- MN558945).

Quality controls to exclude contamination during molecular assays was checked using nuclease free water as non-template controls and negative human plasma from a blood donor as a negative control for the extractions and amplification reactions.

### Post-birth and follow-up

All children were vaccinated within 24 h after birth. For the follow-up 3 years later, the HBsAg positive mothers were contacted via mobile phone and recalled with their children. Only 7/22 (32%) children were brought to the follow-up visit. A whole blood sample was drawn from the children, centrifuged at 3000 rpm (RPM) to separate serum that was then frozen at − 20 °C for 10 months before shipping to the Department of Virology, University of Wuerzburg, Germany, for further serologic and molecular analysis. In Germany, HBsAg testing was performed using HBsAg qualitative II test on 2Architect (Abbott, Ireland), HBV viral load with COBAS® TaqMan® using High Pure System (Roche, Switzerland).

### Statistical analysis

Data were entered into a Microsoft Excel database. Statistical analyses were performed using IBM SPSS Version 25 software. Quantitative variables are expressed as mean ± standard deviation (SD). *P*-values for variables with skewness and kurtosis outside the range of [− 2;+ 2] were calculated using Mann-Whitney U test, *p*-values for skewness and kurtosis within the range of [− 2;+ 2] were calculated using the independent sample T-test. Odds ratios, confidence intervals and *p*-values were calculated using logistic regression. A *p*-value of 0.05 or lower was considered significant. For the models with a binary outcome of zero, 0.5 was added to all numbers to generate a hazard ratio.

## Results

### Patient characteristics

A total of 743 women were recruited into the study. The median age was 26 years (IQR: 31–22 = 9). According to the questionnaire, 42 (5.7%) of the participants self-reported to be HIV positive, while 17 (2.3%) did not know their status. Only 5 (0.7%) patients had an HBV test before.

Most of the participants lived in an urban area (710, 95.7%) and 689 (93.5%) were cohabiting or married. In terms of maximum education level attained, primary education was completed by 314 (42.4%) participants and 389 (52.6%) completed secondary education and/or went to college. Of 741 participants,^1^ 429 (57.9%) were employed, of which 26 (3.5%) were in the health sector. The remaining 312 participants (42.0%) were not employed and/or in a housewife role.

For 271 women (36.5%) it was the first pregnancy. Of those included, 287 women (38.6%) reported they had had only one sexual partner in their life, and the vast majority, 712 (96.1%), reported they had their first sexual contact above the age of 15. Only 14/701 (1.9%) reported receiving at least one dose of HBV vaccination prior to enrolment into the study. The majority, 733 (99.5%), sometimes or never used a condom. Razor blades were shared more often than toothbrushes (21.9% vs. 12.2%). Female genital mutilation was reported in 23 cases (3.1%). Only a small number (4.8%) reported a known history of sexual transmitted infections (STIs). Of 716 participants, 556 (74.8%) had never had any contact with blood containing risk factors such as blood donation, intravenous drugs and/or any infusion or injection (see Table 1).

**Table 1:** Characteristics of participants (*n* = 743) according to HBsAg status

	Total		HBsAg positive		HBsAg negative		***p***-value
	n	(%)		n (%)		n (%)	
Age (years), mean ± SD	26.98 ± 6.03		27.45 ± 5.4		26.97 ± 6.05		0.704
18–20	106	(14.3)	1	(4.5)		105 (14.6)	
21–30	447	(60.2)	16	(72.7)		431 (59.8)	
31–40	172	(23.1)	4	(18.2)		168 (23.3)	
> 40	18	(2.4)	1	(4.5)		17 (2.4)	
HIV status^a^							
HIV positive	42	(5.8)	2	(9.1)		40 (5.7)	0.509
HIV negative	678	(94.2)	20	(90.9)		658 (94.3)	
Residence^b^							
Urban	710	(95.7)	21	(95.5)		689 (95.7)	0.957
Rural	32	(4.3)	1	(4.5)		31 (4.3)	
Marital status^c^							
Single + Divorced + Widowed	48	(6.5)	3	(13.)		45 (6.3)	0.170
Cohabiting + Married	689	(93.5)	19	(86.4)		670 (93.7)	
Education^d^							
Non-formal	37	(5.0)	1	(4.5)	36	36 (5.0)	0.845
Primary	314	(42.4)	9	(40.9)	305	305 (42.5)	
Secondary + College	389	(52.6)	12	(54.5)	377	377 (52.5)	
Work^e^							
Health sector	26	(3.5)	2	(9.1)	24	24 (3.3)	0.080
Employed	403	(54.4)	14	(63.6)	389	389 (54.1)	
Non employed/house wife	312	(42.1)	6	(27.3)	306	306 (42.6)	
Gravida^f^							
One	271	(36.5)	10	(45.5)	261	261 (36.3)	0.199
Two or more	419	(59.4)	12	(54.5)	459	459 (63.7)	
Para^g^							
Zero	299	(40.3)	12	(54.5)	287	287 (39.9)	0.097
One or more	491	(59.7)	10	(45.5)	453	453 (60.1)	
Number of sexual partner							
One	287	(38.6)	7	(31.8%)	280	280 (38.8)	0.505
Two-three	372	(50.1)	12	(54.5)	360	360 (49.9)	
Four or more	84	(11.3)	3	(13.6)	81	81 (11.2)	
Age of first sexual contact^h^							
Under 15	29	(3.9)	0	(0.0)	29	29 (4.0)	0.348
15 or above	712	(96.1)	21	(100)	691	691 (96.0)	
HBV vaccination^i^							
Received at least one vaccination	14	(2.0)	0	(0.0)	14	14 (1.9)	0.507
No vaccination	687	(98.0)	21	(100)	666	666 (97.9)	
History of STIs^j^							
Yes	35	(4.8)	2	(9.1)	33	33 (4.6)	0.334
No	699	(95.2)	20	(90.9)	679	679 (95.4)	
Condom use^k^							
Always	4	(0.5)	0	(0.0)	4	4 (0.6)	0.725
Sometimes + never	733	(99.5)	22	(100)	711	711 (99.4)	
Female genital mutilation^l^							
Yes	23	(3.1)	1	(4.5)	22	22 (3.1)	0.703
No	708	(96.9)	21	(95.5)	687	687 (96.9)	
Sharing toothbrush^m^							
Yes	91	(12.4)	3	(13.6)	88	88 (12.3)	0.856
No	644	(87.6)	19	(86.4)	625	625 (87.7)	
Sharing razor^n^							
Yes	162	(21.9)	3	(13.6)	159	159 (22.1)	0.342
No	578	(78.1)	19	(86.4)	559	559 (77.9)	
History of jaundice^o^							
In the family	73	(9.9)	2	(9.1)	71	(9.9)	0.842
None	668	(90.1)	20	(90.9)	648	(90.1)	
General risk from blood containing procedures^p^							
Risky procedure in the past	161	(22.5)	6	(27.3)	155	(22.3)	0.583
No risky procedure in the past	556	(77.5)	16	(72.7)	540	(77.7)	

^a^23 missing

^b^1 missing

^c^6 missing

^d^3 missing

^e^2 missing

^f^1 missing

^g^1 missing

^h^2 missing

^i^42 missing

^j^9 missing

^k^6 missing

^l^12 missing

^m^8 missing

^n^3 missing

^o^2 missing

^p^26 missing

### Laboratory results

#### Serology results

Of the 743 women tested, 22 (3.0%) were HBsAg positive when screened with the rapid test, which was confirmed using a laboratory-based assay. Of the 22 HBsAg positive samples, 2 (9%) were HBeAg positive.

Umbilical cord blood samples from 15 babies born to HBsAg positive mothers were available. Of these, 5/15 (33%) were tested positive for HBsAg. At 3 years post-birth, a follow-up assessment was carried out on 7 (32%) of the 22 children born to HBsAg positive mothers. The other 15 children were lost to follow-up. Five (71%) of the seven children that were followed up had protective anti-HBs levels (> 10 mIU/ml). One child had anti-HBs level of 7 mIU/ml. One of the seven children was HBsAg positive and also positive for HBeAg, suggesting an established hepatitis B infection.

#### Virologic results

**Fig. 1:**
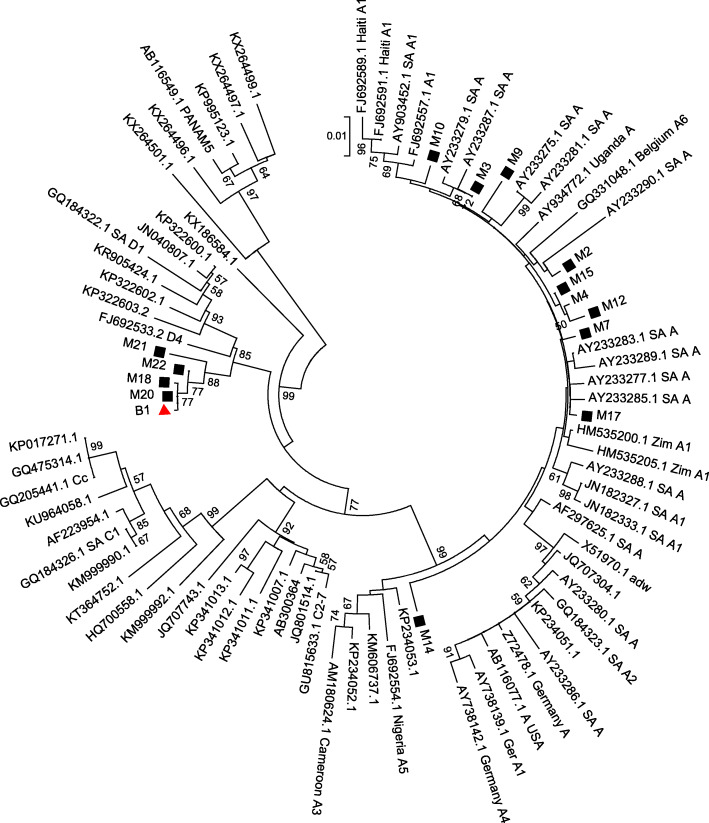
Molecular Phylogenetic analysis by Neighbour Joining method. The evolutionary history was inferred by using the Maxi Neighbour Joining method based on the Tamura-Nei model. Sequences derived from mothers samples are prefixed by M- and annotated with a black square while the single child-derived sequence is denoted as B1 and annotated with a red triangle. Sequences from the study were aligned against other HBV sequences deposited on GenBank

HBV DNA was detectable in 21/22 of the HBsAg positive maternal blood samples. The median viral load among those with detectable HBV DNA was 241 IU/ml (IQR: 83–1730). Of the 14 samples obtained from the pregnant women that were sequenced, 10 (71%) belonged to HBV genotype A while 4 (29%) were genotype D (Fig. 1). Cord blood samples were not tested for HBV DNA. The one HBsAg positive child at 3 years follow-up (indicated as B1 in Fig. 1) had a hepatitis B viral load of 6.3 × 10^7^ IU/ml and was infected with HBV genotype D. Pairwise distance analysis of the virus obtained from the child’s (B1) and mother’s sample (M20 on Fig. 1) on a 593 bp fragment of the polymerase/surface antigen region indicated no base differences per site between the two sequences, indicating similarity. There were no vaccine-escape mutations observed in the child-derived sequence.

#### HBV and socio-demographic characteristics

Logistic regression indicated odds were higher for mothers to be infected with HBV if they sometimes or never used condoms (OR = 3.514, 95% CI = 1.4–8.0). HBsAg positivity was seen in 2/42 (4.8%) of self-reported HIV-infected cases compared to 20/678 (3.0%) of HIV-uninfected cases, Fisher’s exact test *p* = 0.4 (see Table 2).

**Table 2:** Logistic Regression of characteristics and HBV infection (*n* = 743)

	OR	95% CI		***p***-value
Age of the participants (years)				
18–20	1			0.480
21–30	3.898	0.511	29.724	
31–40	2.500	0.276	22.672	
> 40	6.176	0.369	103.494	
HIV status				
HIV positive	1.645	0.371	7.286	
HIV negative	1			0.512
Residence				
Urban	1			0.956
Rural	1.058	0.138	8.124	
Marital status				
Single + Divorced + Widowed	2.351	0.671	8.242	0.182
Cohabiting + Married	1			
Education				
Non-formal	1			0.981
Primary	1.062	0.131	8.629	
Secondary + College	1.146	0.145	9.067	
Work				
Health sector	4.250	0.813	22.204	
Employed	1.835	0.697	4.832	
Non employed/house wife	1			0.194
Gravida				
One	1.466	0.625	3.439	0.380
Two or more	1			
Para				
Zero	1.810	0.772	4.246	0.172
One or more	1			
Number of sexual partners				
One	1			0.791
Two-three	1.333	0.518	3.431	
Four or more	1.481	0.375	5.859	
Age of first sexual contact				
Under 15	1.834	0.750	4.486	0.184
15 or above	1			
HBV vaccination				
Received at least one vaccination	1			
No vaccination	1.069	0.434	2.634	0.884
History of STIs				
Yes	2.058	0.461	9.175	
No	1			0.344
Condom use				
Always	1			
Sometimes + never	3.514	1.382	8.036	0.008
Female genital mutilation				
Yes	1.487	0.191	11.557	0.705
No	1			
Sharing toothbrush				
Yes	1.121	0.325	3.867	0.856
No	1			
Sharing razor				
Yes	1			
No	1.801	0.526	6.165	0.348
History of jaundice				
Family or myself	1			
None	1.096	0.251	4.785	0.903
General risk from blood contact				
Risky procedure in the past	1.307	0.503	3.396	0.5833
No risky procedure in the past	1			

## Discussion

Despite the reported higher intermediate prevalence of hepatitis B infection in Tanzanian people [4], our study cohort, pregnant women attending antenatal care or delivering at a tertiary hospital in Mwanza showed a low intermediate prevalence of CHB with 22/723 (3%) being HBsAg positive. This could be due to recruitment from a tertiary hospital which induces a social selection bias of the participants. The high percentage of completed secondary or higher education (52.6%), compared to the national net enrollment rate of secondary education of 23% in 2017 [26], is also an indicator of possible social selection bias. However, another study found a comparable prevalence of HBsAg among women delivering in primary health care settings in the area of Mwanza [5], which typically do not charge for user fee. This may indicate a lower prevalence in this area compared to the rest of Tanzania where a prevalence of 7.17% has been reported [4]. Given a lack of standard therapy for patients with chronic HBV infection accessible in Tanzania [10], even this low percentage presents a problem for patients, relatives and the health-care system alike. Hepatitis B prevalence disparities among pregnant women between regions within a country have also been reported in other SSA countries. In Kenya, where the national hepatitis B prevalence among pregnant women was 9.3%, intradistrict HBV prevalence disparities ranged between 4.3 and 17.8% [27]. Similarly, in Nigeria large local differences in HBV prevalence have been observed [28].

Factors which have been found to be associated with HBsAg positivity in other Tanzanian and African studies include multigravidity, urban residence, younger age (15–24 years), presence of other sexually transmitted diseases, history of blood transfusions, oral contraceptives use, anemia, body tattooing and unsafe injections [5, 29, 30]. In our study, only the characteristic of no or sometimes condom use shows a statistically significant higher association (OR = 3.514, *p* = 0.008) with a positive HBsAg result. However, given only four participants indicated the use of condoms as ‘always’, our results need to be interpreted with care. Additionally, as we were looking at pregnant women, it is unlikely that the condom use was consistent and there might be a bias in the classification, as well as the responses. The lack of significance for other characteristics may be due to the small number of HBsAg positive cases that could conceal significant results in the logistic regression. Therefore, additional studies with larger sample sizes are necessary to confirm these results.

Two women were positive for HBeAg, which usually indicates high viral replication. One of these women (see Table 2; number 20) had the highest viral load of 25.9 × 10^7^ IU/ml. In the follow-up her child was positive for HBsAg, HBeAg and anti-HBc and had a viral load of 6.3 × 10^7^ IU/ml. Even though no other sample from this child is available, it is very likely that these results indicate CHB resulting from a vertical transmission. Indeed, the phylogenetic relationship and pairwise distance analysis showed identical nucleotide bases across the sequenced polymerase region of the virus derived from the mother’s and the child’s blood. It could be argued that the recommendation to use tenofovir in highly-viraemic HBV-infected pregnant women within the third trimester might have helped to prevent infection in the child [31]. Unfortunately, tenofovir was not licensed in Tanzania at the time of enrollment for this study. Administration of vaccine alone was not enough to prevent infection in the child. Our results show that the strain that infected the child was amenable to vaccination because no mutations that may be associated with vaccine escape were detected. This therefore rules out the possibility that the child could have been infected due to vaccine failure.

Two other mothers had a viral load > 2000 IU/ml which represents an indication for treatment for CHB [21]. Both of their children had HBsAg positive cord blood samples at birth but were unfortunately lost to follow-up. Follow-up was only possible for one child with HBsAg positive and six with HBsAg negative umbilical cord blood samples at birth. At follow-up, 5/7 had protective anti-HBs levels and no anti-HBc indicating successful vaccination and no exposure to natural infection. Many children born to HBV-infected mothers were lost to follow-up because we could not contact the mothers due to change of mobile phone numbers, relocation to other areas away from the study site and in some cases, inability to come to the hospital for other reasons.

The observation that 5 out of 15 cord blood samples were positive for HBsAg needs to be interpreted with caution, because of the high risk of contamination with maternal blood during delivery. This could explain one child having HBsAg positive umbilical cord blood, but no HBsAg positivity in the follow-up. The high workload of the health-care workers also led to the low number of women recruited for the study (compared to the number of deliveries taking place) and missing cord blood samples. Additionally, a presence of HBsAg in cord blood does not necessarily reflect an infection with HBV and merely indicates an exposure, as a recent study by Liu et al. has shown [32].

The HBV genotype varies according to the geographic region: genotype A is mainly found in southern, eastern and central Africa while genotype D is predominant in northern and genotype E in western Africa [33]. Our sequencing results and phylogenetic analysis by maximum likelihood showed a high prevalence of genotype A (71,4%), and a low prevalence of genotype D (28,6%) in line with previous findings in Tanzania [34]. Genotype A may have a more severe disease outcome rather than D, but further studies are needed to investigate this [33, 35].

A methodological limitation of our study is the use of HBsAg rapid testing as the only screening method for CHB, as it misses occult HBV infections. Due to mutations of the HBV surface antigen gene occult infections cannot be detected by common HBsAg tests [36]. The method of choice to detect occult HBV infections is real time PCR but this was not available at the study site. Allain et al recently highlight the prevalence of occult HBV infection in general population ranges between 1:100–1000 in high prevalence areas of West Africa and Asia [37]. Therefore, we estimate that missed cases of occult HBV infection did not considerably influence our study results. The missing values of some of the characteristics of participants (Table 1), as well as the high number of the loss to follow-up are another limitation of the study. A more comprehensive collection of data could have allowed a better, more precise analysis.

Another general limitation of this assessment is the social desirability bias, especially in the context of sexual behavior. Another limitation is the high possibility of recall bias, especially for questions on previous injections and other medical treatments. This means that the results of the analysis of the patient characteristics need to be interpreted with caution, because of possible low or wrong answers (e.g. on the number of sexual partners, the age of first sexual contact, number of medical interventions). The specific study group does not allow an extrapolation to pregnant women in general in Tanzania.

Conducting a study on Hepatitis B infection in a setting where no medical treatment is available brings up ethical dilemmas that need to be considered cautiously. Even though it was not possible to provide the women themselves with treatment for their infection, the diagnosis may have increased their awareness for this infection and thereby possibly contributed to the reduction of transmission.

## Conclusion

The HBsAg prevalence of 3% among 743 pregnant women in this study conducted at a tertiary hospital in Mwanza was lower compared to other studies on pregnant women [5–8], or the general population in Tanzania [4]. In the follow-up 3 years later only one (14%) of seven children showed evidence of chronic HBV infection. The mother of the child had a high viral load (25.9 × 10^7^ IU/ml) and was positive for HBeAg and is likely to have been the source of infection in the child, as revealed by the similarity of their viral sequences. With no standard therapy for CHB accessible in Tanzania, even this comparably low number of cases indicate a problem for the women and their children. We suggest screening of pregnant women and newborn vaccination should be implemented to prevent newborns from infection.

## Supplementary information

**Additional file 1: Table S1.** Oligonucleotide primers used for the nested PCR amplification of the HBV polymerase gene. **Table S2.** HBsAg positive participants and laboratory results (*n* = 22).

## Data Availability

The datasets used and/or analysed during the current study are available from the corresponding author on reasonable request.

## Abbreviations

anti-HBc: Hepatitis B core antibody
anti-HBe: Hepatitis B envelope antibody
anti-HBs: Hepatitis B surface antibody
BMC: Bugando Medical Centre
CHB: Chronic hepatitis B
CRF: Case report form
CUHAS: Catholic University of Health and Allied Sciences
DNA: Desoxyribonucleic acid
ELISA: Enzyme-linked immunosorbent assay
HBeAg: Hepatitis B envelope antigen
HBsAg: Hepatitis B surface antigen
HBV: Hepatitis B virus
HDV: Hepatitis D virus
HCC: Hepatocellular carcinoma
IQR: Interquartile range
PCR: Polymerase chain reaction
PMTCT: Prevention from mother to child transmission
SSA: Sub-Sahara Africa
STIs: Sexually transmitted infections
WHO: World Health Organization

## Footnotes

1 Information from 2 participants were missing.
