# Supplementary material for: Prevalence of chronic HBV infection in pregnant woman attending antenatal care in a tertiary hospital in Mwanza, Tanzania: a cross-sectional study
Source: BMC Infect Dis. 2020 Jun 5;20:395. doi: 10.1186/s12879-020-05096-2 (PMC7275503; doi:10.1186/s12879-020-05096-2)
Supplement: Supplementary file 1 — Additional file 1: Table S1. Oligonucleotide primers used for the nested PCR amplification of the HBV polymerase gene. Table S2. HBsAg positive participants and laboratory results (n = 22). [file 12879_2020_5096_MOESM1_ESM.docx]

Supplementary files

**Table 1: Oligonucleotide primers used for the nested PCR amplification of the HBV polymerase gene and the**

| **Primer** | **Sequence** |
| --- | --- |
| **First round PCR** | |
| HBV Z | 5' AGCCCTCAGGCTCAGGGCATA 3’ |
| HBV 3 | 5' CGTTGCCKDGCAACSGGGTAAAGG 3’ |
| *Second round PCR* | |
| HBV M | 5’ GACACA CTTTCCAATCAATNGG 3’ |
| HBV P | 5’ TCATCCTCAGGCCATGCAGT 3’ |
| *Sequencing PCR* | |
| HBV P | 5' TCA TCC TCA GGC CAT GCA GT |
| HBV M | 5' GAC ACA CTT TCC AAT CAA TNG G |
| HBV H | 5’ TAT CAA GGA ATT CTG CCC GTT TGT CCT |
| HBV N | 5’ ACTGAGCCAGGAGAAACGGACTGAGGC |

| **Table 2. HBsAg positive participants and laboratory results (n=22)** | | | | | |  |  |  |  |  |
| --- | --- | --- | --- | --- | --- | --- | --- | --- | --- | --- |
|  |  |  |  |  |  |  |  | **Child follow-up** | | |
| **mother** | **HIV positive** | **anti-HBe** | **HBeAg** | **genotype** | **HBV viral load (IU/ml)** | **HBsAg in cord blood of the child** |  | **HBsAg** | **anti-HBs*** | **HBcAb** |
| 1 | no | positive | negative | ND | 89.5 | negative |  | negative | positive | negative |
| 2 | no | positive | negative | A | 144 | negative |  | undetermined | undetermined | undetermined |
| 3 | no | positive | negative | A | 288 | N/A |  | undetermined | undetermined | undetermined |
| 4 | yes | positive | negative | ND |  | N/A |  | undetermined | undetermined | undetermined |
| 5 | no | positive | negative | ND | 48.4 | negative |  | undetermined | undetermined | undetermined |
| 6 | yes | positive | negative | ND | 162 | positive |  | negative | positive | negative |
| 7 | no | positive | negative | A | 2160 | positive |  | undetermined | undetermined | undetermined |
| 8 | no | positive | negative | N/A | 61.5 | negative |  | undetermined | undetermined | undetermined |
| 9 | no | positive | negative | A | 241 | N/A |  | undetermined | undetermined | undetermined |
| 10 | no | positive | negative | A | 1870 | negative |  | negative | positive | negative |
| 11 | no | positive | negative | ND | 27.4 | positive |  | undetermined | undetermined | undetermined |
| 12 | no | negative | positive | A | 570 | N/A |  | negative | negative | negative |
| 13 | no | positive | negative | A | 322 | negative |  | negative | positive | negative |
| 14 | no | positive | negative | A | 108 | negative |  | undetermined | undetermined | undetermined |
| 15 | no | positive | negative | A | 1720 | negative |  | undetermined | undetermined | undetermined |
| 16 | no | positive | negative | ND | 57.8 | negative |  | undetermined | undetermined | undetermined |
| 17 | no | positive | negative | A | 1020 | negative |  | undetermined | undetermined | undetermined |
| 18 | no | positive | negative | D | 2890 | positive |  | undetermined | undetermined | undetermined |
| 19 | no | positive | negative | ND | 411 | positive |  | undetermined | undetermined | undetermined |
| 20 | no | negative | positive | D | 259000000 | N/A |  | positive | negative | positive |
| 21 | no | positive | negative | D | 76.0 | N/A |  | undetermined | undetermined | undetermined |
| 22 | no | positive | negative | D | 176 | N/A |  | negative | positive | negative |
|  |  |  |  |  |  |  |  |  | *>10 IU/ml | |
